# Academics’ and clinicians’ perspectives on telehealth integration in Saudi rehabilitation education

**DOI:** 10.1186/s12909-026-08639-4

**Published:** 2026-01-24

**Authors:** Rania Alkahtani, Nisreen Naser Al Awaji, Ahmad S Alanazi, Eman M. Mortada, Maha Aldera, Ghadah S. Aljarboa

**Affiliations:** 1 Department of Health Communication Sciences, College of Health and Rehabilitation Sciences, Princess Nourah bint Abdulrahman University, P.O. Box 84428, Riyadh, 1167 Saudi Arabia; 2 Department of Physical Therapy, college of Medical rehabilitation Sciences, Taibah University, Madinah , 42353 Saudi Arabia; 3 Family and Community medicine department, College of Medicine, Princess Nourah Bint Abdulrahman University, P.O. Box 84428, Riyadh, 11671 Saudi Arabia

**Keywords:** Telehealth, education, Rehabilitation, Curriculum, Saudi arabia

## Abstract

**Background:**

Telehealth is increasingly embedded within healthcare delivery, yet its implementation in rehabilitation education in Saudi Arabia remains limited and inconsistently implemented. Understanding the perspectives of academics and clinicians is essential for informing curriculum reform. This study examined perceptions of telehealth teaching and training among rehabilitation academics and clinicians in Saudi Arabia.

**Methods:**

A cross-sectional online survey using convenience sampling was completed by academics (*n* = 56) and clinicians (*n* = 144) across rehabilitation disciplines. Participants who were currently teaching or practicing in Saudi Arabia were eligible. Descriptive statistics and Chi-square tests were used to compare familiarity, use, and attitudes toward telehealth education.

**Results:**

Most respondents (> 75%) supported incorporating telehealth into undergraduate rehabilitation curricula and endorsed hands-on training for developing telehealth competencies. Academics expressed significantly stronger support for curricular integration than clinicians (χ²=12.49, *p* = .002). Prior exposure to telehealth was significantly associated with greater support for inclusion (*p* < .05). The most frequently cited barriers were that telehealth course inclusion had not been previously discussed and limited awareness of supporting research, whereas technological infrastructure was reported less often.

**Conclusions:**

These findings indicate broad support for telehealth education among rehabilitation professionals in Saudi Arabia, while highlighting a gap between perceived importance and curricular readiness shaped by prior exposure and resource availability. The results highlight the need for faculty development, investment in digital infrastructure, and collaborative academic–clinical planning to ensure telehealth competencies are systematically embedded into rehabilitation training. Collectively, this study provides foundational evidence to inform national curriculum reform and digital health workforce development aligned with Saudi Arabia’s digital health transformation efforts.

**Supplementary Information:**

The online version contains supplementary material available at 10.1186/s12909-026-08639-4.

## Introduction

Telehealth has become an increasingly integral component of healthcare delivery, enabling patients to access clinical services and professional consultations without geographic or logistical constraints [1]. By reducing barriers related to provider availability and transportation, telehealth has demonstrated substantial potential to improve accessibility and health outcomes, particularly in underserved or remote settings [2, 3]. In Saudi Arabia, telehealth expansion aligns with Saudi Vision 2030, which prioritizes advanced technologies to enhance healthcare efficiency and access [4, 5]. National initiatives such as the Seha Virtual Hospital have broadened specialist service reach across the Kingdom [6], underscoring strong infrastructural readiness and the parallel need for a workforce equipped with competencies required for remote care delivery.

International health-professions programs, particularly in the United States, Canada, and Australia, have begun embedding telehealth competencies into their curricula using digital-pedagogy frameworks such as Technological Pedagogical and Content Knowledge (TPACK), Substitution, Augmentation, Modification, and Redefinition (SAMR), and the Community of Inquiry model (CoI) [7–9]. In contrast, Saudi rehabilitation programs have made limited progress in integrating similar frameworks, despite rapid national advances in digital healthcare through initiatives such as Seha Virtual Hospital. Existing educational efforts in the Kingdom remain concentrated in medicine and nursing, with minimal emphasis on telehealth training in allied-health and rehabilitation disciplines [10, 11]. This disparity highlights the need for theory-informed approaches to guide telehealth curriculum integration in Saudi rehabilitation education. Recent international work further reinforces this need. Edirippulige et al. [12] demonstrated that telehealth competencies must be intentionally embedded into health-professions curricula through scaffolded skill development, simulated encounters, and explicit assessment of digital communication and clinical decision-making. Such competency-based approaches provide a useful model for guiding curriculum development.

Telehealth encompasses synchronous and asynchronous modalities that have demonstrated comparable outcomes to in-person care while improving access and continuity [13–16]. Despite these advantages, successful implementation depends on clinicians’ digital literacy and their ability to adapt assessment and intervention strategies to virtual environments [17, 18], underscoring the importance of structured educational preparation. The Technology Acceptance Model (TAM) provides a relevant foundation for understanding educators’ and clinicians’ readiness to adopt telehealth. TAM posits that perceived usefulness and perceived ease of use shape attitudes toward technology and ultimately predict usage behavior [19]. These constructs are directly applicable to both clinical and telehealth integration.

Globally, institutions have begun integrating telehealth into healthcare curricula to prepare students for evolving models of care [20]. The Association of American Medical Colleges (AAMC) has articulated competencies related to digital literacy, ethics, communication, and data security [21], while professional rehabilitation bodies such as the American Academy of Audiology (AAA) and the American Speech-Language-Hearing Association (ASHA) advocate for the inclusion of telehealth training in professional preparation programs [22, 23]. These standards collectively underscore the importance of integrating telehealth education to ensure workforce readiness.

Integrating telehealth into healthcare curricula requires grounding in established digital-pedagogy frameworks. TPACK emphasizes the integration of technological, pedagogical, and disciplinary knowledge, while the SAMR and CoI models support meaningful transformation of learning activities and the development of cognitive, social, and teaching presence in digital environments [7–9, 24–26]. Together with TAM, these frameworks provide a coherent conceptual foundation for designing telehealth-enhanced rehabilitation curricula. In this study, these frameworks (TAM, TPACK, SAMR, and CoI) were used to conceptually inform understanding of curriculum integration and to understand educators’ and clinicians’ perspectives, rather than to structure theory-driven measurement or scale development.

Recent analyses of digital transformation in health sciences education further emphasize the need for pedagogically aligned and thoughtfully designed technology integration to prepare students for digitally mediated clinical environments [10, 11]. These insights reinforce the importance of incorporating telehealth competencies into rehabilitation training programs.

In Saudi Arabia, research on telehealth has predominantly focused on medical and nursing perspectives, with limited exploration of rehabilitation specialties. Recent national studies have examined healthcare professionals’ knowledge and attitudes toward telehealth [27, 28], yet none have assessed the readiness of rehabilitation educators and clinicians to integrate telehealth teaching and training into undergraduate programs. Understanding their perspectives is essential to align educational outcomes with current and future clinical demands.

Despite rapid national expansion of telehealth infrastructure, a disconnect persists between service-level digital transformation and the educational preparation of rehabilitation students. Most undergraduate programs lack structured telehealth training, competency development, and exposure to digital clinical tools, leaving graduates underprepared for hybrid or virtual care models. Addressing this gap requires a clear understanding of how educators and clinicians perceive telehealth integration, the barriers they anticipate, and the supports required for curriculum redesign. Thus, this study aimed to comparatively and exploratorily examine how academics and clinicians experiences and professional roles shape their attitudes toward telehealth integration into undergraduate curricula in Saudi Arabia. Specifically, it explored differences between academics and clinicians in (1) familiarity with and use of telehealth, (2) attitudes toward incorporating telehealth teaching and training in rehabilitation programs, and (3) perceived barriers and facilitators influencing curriculum integration.

## Methods

### Study design, participants, and recruitment

This cross-sectional survey was conducted in Saudi Arabia among two groups: (1) academic staff teaching in rehabilitation-related programs at Saudi universities and (2) clinicians practicing in audiology, speech-language pathology, physiotherapy, occupational therapy, or respiratory therapy. A convenience sampling approach was used to recruit participants; therefore, the sample reflects the portion of the target population that could be reached through professional networks and social media platforms. Individuals whose academic or clinical roles did not have a rehabilitation focus were excluded. Non-practicing academics were eligible provided they were teaching in rehabilitation-related programs. Participation was voluntary and anonymous, and electronic informed consent was obtained prior to survey completion.

### Instruments

No existing validated telehealth questionnaires capture curriculum-related perspectives, institutional readiness, or program-level factors in rehabilitation education, particularly across multiple disciplines and within the Saudi context. Therefore, self-developed instruments, one for academics and another for clinicians, were developed based on a literature review, to address the study aims. The academics’ questionnaire (see Appendix A) comprised 19 items organized into six sections: demographic and professional information, familiarity with telehealth, perceived usefulness and attitudes, utilization of telehealth in clinical practice, teaching telehealth, and their future inclusion plans, in addition to their perceived barriers. The clinicians’ questionnaire (see Appendix B) included 18 items grouped into four domains: demographic and professional characteristics, familiarity and use of telehealth, systems used, perceived usefulness, and attitudes toward teaching telehealth to students. The theoretical frameworks referenced in this study (e.g., TAM, TPACK, SAMR, CoI) were used solely to understand educators’ and clinicians’ perspectives, as the questionnaires were purposefully designed for exploratory assessment rather than theory-driven measurement.

Content validity was established through expert review by five rehabilitation specialists, who evaluated each item for clarity, relevance, and alignment with the study objectives. Based on their feedback, several items were refined for clarity (e.g., ‘Do you have clinical experience?’ was revised to ‘Do you practice clinically?’ and ‘Is the term telehealth familiar to you?’ was revised to ‘Are you familiar with the term telehealth?’). In addition, one item addressing perceived reasons for unwillingness to include telehealth in rehabilitation curricula was added to better capture institutional and contextual barriers.

The questionnaires were subsequently piloted with 15 participants, who confirmed that the items were clear and comprehensible; no further modifications were required. Pilot responses were retained and included in the final analysis to avoid unnecessary reduction of the sample size.

The internal consistency of the instrument was assessed using Cronbach’s alpha for the three subscales (perception, use, and barriers), with coefficients of 0.76, 0.69, and 0.63, respectively. While the perception and use subscales demonstrated acceptable internal consistency, the barriers subscale demonstrated a lower internal consistency (α = 0.63); however, this value is considered acceptable for exploratory research involving heterogeneous, practice-based items. Nevertheless, findings related to this subscale should be interpreted with caution, and future studies may refine these items to improve reliability.

### Data collection and analysis

Data were collected electronically using a secure online platform. Each participant completed only one version of the questionnaire corresponding to their professional role. The survey link was disseminated through professional societies and established professional networks within rehabilitation disciplines in Saudi Arabia; no direct email invitations were sent. The survey platform required responses to all mandatory items, ensuring data completeness for the variables analyzed.

Data were analyzed using SPSS (version 20, IBM Corp., Armonk, NY, USA). Descriptive statistics summarized participant characteristics and responses. Chi-square and Fisher’s exact tests were used to compare responses between academics and clinicians, with statistical significance set at *p* ≤ .05. These tests were selected due to the categorical nature of the variables. For each Chi-square test, effect sizes were calculated to assess the practical significance of the associations using both Phi (φ) was used for 2 × 2 tables and Cramér’s V whenever applicable.

### Ethical considerations

Ethical approval was obtained from the Institutional Review Board of Princess Nourah bint Abdulrahman University (IRB No. 21-0272). Informed consent was obtained digitally at the beginning of the survey: participants were provided with an overview of the study objectives, investigator contact information, and assurances regarding voluntariness and confidentiality, and were required to indicate their agreement to participate before accessing the survey questions. Participants who did not provide consent were automatically exited from the survey.

Data were collected through a secure, encrypted online survey platform. No identifying information (e.g., names, email addresses, or IP addresses) was recorded, and institutional affiliations were captured only as non-identifiable categorical options. All responses were stored anonymously, and only aggregated data were analyzed to ensure confidentiality and protect participants’ privacy, in accordance with institutional ethical guidelines and applicable Saudi data privacy regulations. Participation was entirely voluntary, and participants could withdraw from the survey at any point before submission without providing any justification.

## Results

Of 151 clinicians who accessed the survey, 144 completed it and provided analyzable data. The demographic characteristics of clinicians are summarized in Table 1. Females and Saudi nationals comprised the majority, reflecting workforce composition in the clinical sector. Most clinicians were specialists working in public hospitals or clinics, highlighting the prominence of these settings in the sample. Notably, just over one-third had experience collaborating in university teaching, suggesting that academic involvement among clinicians remains relatively limited.

**Table 1:** Demographic and professional characteristics of clinician respondents (*n* = 144)

Characteristics	*N*	%
Age groups		
21–30	54	37.5
31–40	48	33.3
41–50	37	25.7
51–60	4	2.8
61–70	1	0.7
Gender		
Male	53	36.8
Female	91	63.2
Nationality		
Not Saudi	19	13.2
Saudi	125	86.8
Specialty		
Audiology	44	30.6
Speech language pathology	36	25.0
Nursing	2	1.4
Physiotherapy	8	5.6
Occupational therapy	41	28.5
Respiratory therapy	11	7.6
Low Vision optometrist	1	0.7
Academic rank		
Specialist	84	58.3
Senior specialist	49	34.0
Consultant	11	7.6
Place		
Public hospital/clinic	68	47.2
Private hospital/clinic	44	30.6
Both public/private	29	20.1
Ministry of Health	3	2.1
Years of Experience(years)		
< 2	21	14.6
2–5	40	27.8
6–10	33	22.9
> 10	50	34.7
Have you ever collaborated to teach any course at a university?		
No	92	63.9
Yes	52	36.1

Of 57 academics who accessed the survey, 56 completed it and provided analyzable data. The majority were female and Saudi nationals. Most respondents were physiotherapists, and nearly half were affiliated with Taibah University. Around two-thirds reported active clinical practice, either within university clinics or externally (Table 2).

**Table 2:** Demographic and professional characteristics of academic respondents (*n* = 56)

Characteristics	*N*	%
Age groups		
21–30	8	14.3
31–40	29	51.8
41–50	18	32.1
51–60	1	1.8
Gender		
Male	12	21.4
Female	44	78.6
Nationality		
Not Saudi	8	14.3
Saudi	48	85.7
University		
Taibah	27	48.2
Princess Nourah bint Abdulrahman University	12	21.4
King Saud University	12	21.4
King Abdulaziz University	2	3.6
King Khalid University	1	1.8
Specialty		
Audiology	10	17.9
Speech language pathology	9	16.1
Physiotherapy	21	37.5
Occupational therapy	7	12.5
Respiratory therapy	5	8.9
Low Vision optometrist	2	3.6
Cardiopulmonary physical therapy	1	1.8
Academic rank		
Lecturer	24	42.9
Assistant Professor	24	42.9
Associate Professor	7	12.5
Professor	1	1.8
Years of Experience (years)		
< 2	16	28.6
2–5	9	16.1
6–10	16	28.6
> 10	15	26.8
Do you practice clinically? (either at the university’s clinics or at an outside clinic)?		
No	20	35.7
Yes	36	64.3

A Chi-square test was conducted to examine differences in clinicians’ use of telehealth based on whether they had collaborated in teaching a university course (Table 3). The analysis revealed a statistically significant difference among clinicians who had presented a talk about telehealth (χ² (1) = 4.70, *p* = .04), with a small effect size (φ = 0.18). This suggests that clinicians who participate in educational activities related to telehealth are slightly more likely to have delivered talks on the topic. A significant difference was also found among clinicians who used telehealth to deliver services in their clinics (χ² (2) = 5.77, *p* = .05), accompanied by a moderate effect size (Cramér’s V = 0.22). This indicates that clinicians who engage in collaborative teaching are more likely to incorporate telehealth into their clinical practice. These findings suggest that involvement in academic collaboration may be associated with greater engagement in telehealth-related educational and clinical activities, although the strength of these associations’ ranges from small to moderate.

**Table 3:** Differences in telehealth use among clinician respondents based on their collaboration in teaching theoretical or practical university courses

Characteristics	Have you ever collaborated to teach any course either theoretical or practical at a university?		Total *N* (%)	*P* value	Φ/ Cramer’s V
	No *N* (%)	Yes *N* (%)			
Do you feel comfortable using technology?					
No	4 (4.3)	3 (5.8)	7 (4.9)	0.70	0.03
Yes	88 (95.7)	49 (94.2)	137 (95.1)		
Are you familiar with the term “telehealth”?					
No	6 (6.5)	4 (7.7)	10 (6.9)	0.75	0.02
Yes	86 (93.5)	48 (92.3)	134 (93.1)		
Have you ever attended a lecture, program or workshop on telehealth?					
No	49 (53.3)	22 (42.3)	71 (49.3)	0.23	0.11
Yes	43 (46.7)	30 (57.7)	73 (50.7)		
Have you ever presented a talk about telehealth?					
No	75 (81.5)	34 (65.4)	109 (75.7)	0.04*	0.18
Yes	17 (18.5)	18 (34.6)	35 (24.3)		
Have you conducted research related to telehealth?					
No	79 (85.9)	38 (73.1)	117 (81.2)	0.08	0.16
Yes	13 (14.1)	14 (26.9)	27 (18.8)		
Do you use telehealth to deliver services in your clinic?					
No	60 (65.2)	25 (48.1)	85 (59.0)	0.05*	0.22**
Yes, sometimes	26 (28.3)	25 (48.1)	51 (35.4)		
Yes, regularly	6 (6.5)	2 (3.8)	8 (5.6)		
Do you support the inclusion of telehealth as a course to be taught in the curriculum of Rehabilitation programs?					
No	15 (16.3)	4 (7.7)	19 (13.2)	0.20	0.12
Yes	77 (83.7)	48 (92.3)	125 (86.8)		
Total	92 (63.9)	52 (36.1)	144 (100.0)		

* P-value is statistically significant at ≤ 0.05

** Cramér’s V is used where applicable

Φ indicates Phi as the effect size for 2 × 2 tables

**Fig. 1:**
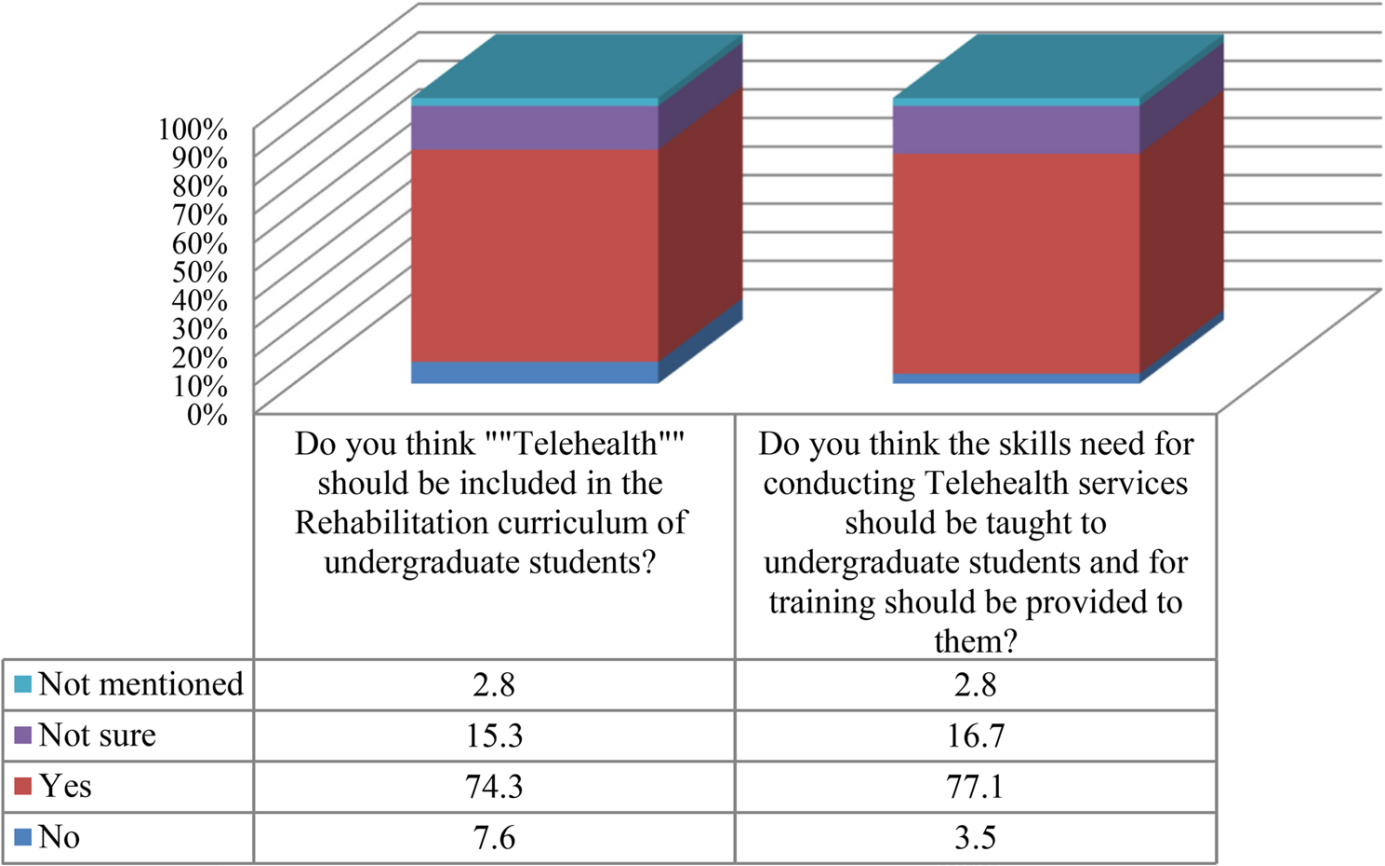
Opinions of clinician respondents regarding telehealth and the skills required to deliver it

There was no statistically significant association between clinicians’ previous collaboration in teaching university courses and their belief that telehealth should be included in undergraduate rehabilitation curricula (χ²(3) = 5.33, *p* = .149). The effect size was small (φ = 0.19), indicating that teaching collaboration explained only a limited proportion of the variability in responses. Nevertheless, most clinicians (74.3%) supported telehealth in the curriculum (Fig. 1). Similarly, teaching collaboration was not significantly associated with clinicians’ views on whether telehealth service-delivery skills and training should be taught to undergraduate students (χ²(3) = 1.26, *p* = .738). The effect size was very small (φ = 0.09), suggesting almost no practical relationship. Support for teaching telehealth skills remained high across groups (77.1%) (Fig. 1).

Academics’ use of telehealth was compared based on their prior experience teaching telehealth-related topics (Table 4). The results indicate meaningful differences across several indicators. Academics who had attended a lecture, program, or workshop on telehealth were significantly more likely to engage with telehealth-related activities (χ² (1) = 7.74, *p* = .01), with a moderate effect size (φ = 0.37). This suggests that exposure to telehealth training or professional development is associated with a greater likelihood of telehealth engagement.

Similarly, those who used telehealth in their own clinical practice differed significantly based on whether they had taught telehealth-related content (χ² (2) = 6.50, *p* = .04), with a moderate effect size (Cramér’s V = 0.37). This indicates that academics who actively use telehealth are also more likely to incorporate it into their teaching.

**Table 4:** Differences in telehealth use among academic respondents based on whether they have taught a telehealth-related course or topic

Characteristics	Have you ever taught a course/topic related to telehealth?		Total *N*. (%)	*P* value	Φ/Cramer’s V
	No *N* (%)	Yes *N* (%)			
Are you familiar with the term “telehealth”? ^#^					
No	5 (13.5)	1 (5.3)	6 (10.7)	0.65	0.13
Yes	32 (86.5)	18 (94.7)	50 (89.3)		
Have you ever attended a lecture, program or workshop on telehealth?					
No	24 (64.9)	5 (26.3)	29 (51.8)	0.01*	0.37
Yes	13 (35.1)	14 (73.7)	27 (48.2)		
Do you practice clinically? (either at the university’s clinics or at an outside clinic)					
No	13 (35.1)	7 (36.8)	20 (35.7)	1	0.02
Yes	24 (64.9)	12 (63.2)	36 (64.3)		
Do you use telehealth in your practice?					
No	20 (54.1)	5 (26.3)	25 (44.6)	0.04*	0.34**
Yes, sometimes	4 (10.8)	7 (36.8)	11 (19.6)		
Yes, regularly	13 (35.1)	7 (36.8)	20 (35.7)		
Have you conducted research related to telehealth?					
No	32 (86.5)	12 (63.2)	44 (78.6)	0.08	0.26
Yes	5 (13.5)	7 (36.8)	12 (21.4)		
Total	37 (66.1)	19 (33.9)	56 (100.0)		

* P-value is statistically significant at ≤ 0.05

** Cramér’s V is used where applicable; Φ indicates Phi as the effect size for 2 × 2 tables

# Fisher’s exact test was used for this item

**Fig. 2:**
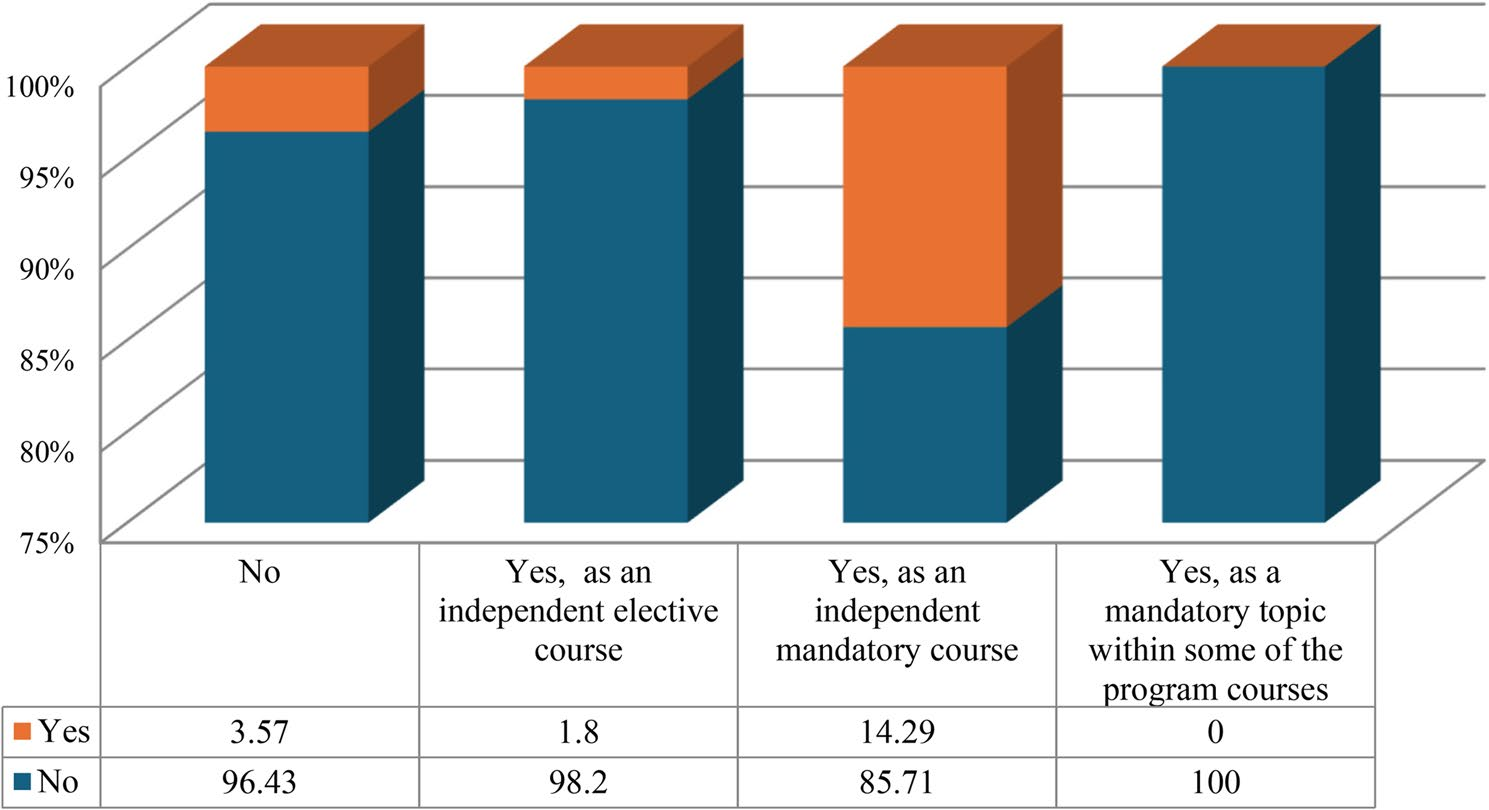
Presence of an academic course in telehealth applications within the programs of respondent academics

**Fig. 3:**
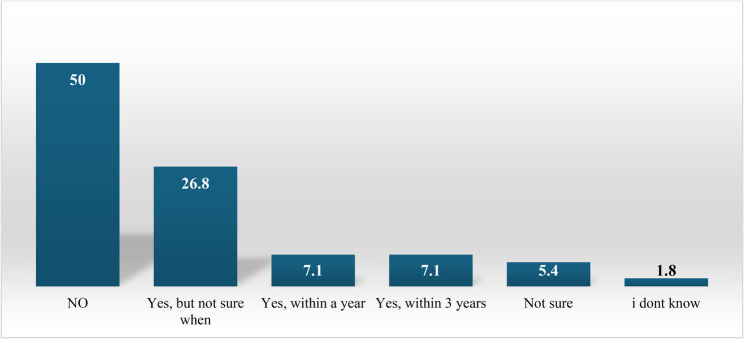
Responses of academic respondents regarding plans to include telehealth teaching and training in their program curricula (*n* = 56)

Figure 2 shows that about 14% of the respondents reported that their program includes an academic course in telehealth as an independent mandatory course. Moreover, approximately 27% of the academics indicated that their programs plan to include telehealth teaching and training in the curriculum but are uncertain about the timeline (Fig. 3).

**Fig. 4:**
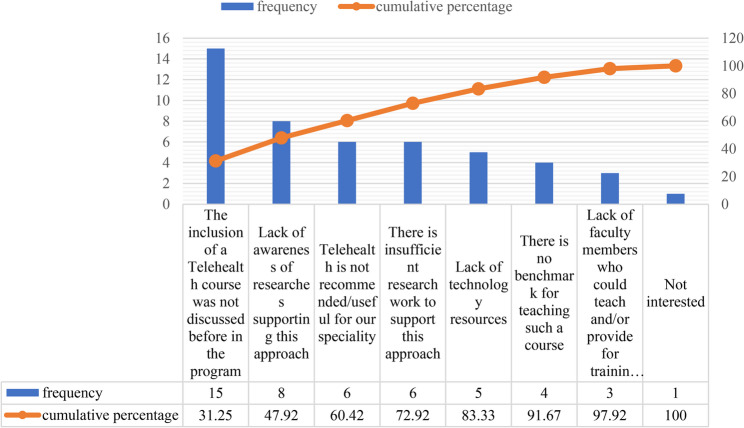
Pareto chart illustrating the reasons cited by academic respondents for their unwillingness to include telehealth in rehabilitation program curricula

Figure 4 presents a Pareto analysis of the reasons cited by academic respondents for their reluctance to include telehealth in rehabilitation program curricula. The distribution of responses demonstrates that a small number of key barriers account for the majority of the reported concerns. The most frequently cited reason was that the inclusion of a telehealth course had not been previously discussed (15 responses; 31.25%). When combined with the second most common barrier, lack of awareness of research supporting this approach (8 responses; cumulative 47.92%), these two factors alone represent nearly half of all concerns. These findings suggest that institutional communication and limited exposure to telehealth-related evidence are the primary contributors to hesitation among academics.

Two additional factors, the perception that telehealth is not recommended or useful for their specialty (6 responses; cumulative 60.42%) and insufficient research evidence to support telehealth (6 responses; cumulative 72.92%), further highlight uncertainties regarding the applicability and strength of the evidence base for telehealth within rehabilitation education. In contrast, factors such as lack of technological resources (5 responses; cumulative 83.33%), absence of benchmarks for teaching telehealth (4 responses; cumulative 91.67%), lack of qualified faculty to deliver or train on such a course (3 responses; cumulative 97.92%), and lack of interest (1 response; 100%) were reported less frequently and contributed minimally to overall resistance. Overall, the Pareto analysis indicates that the predominant barriers are related to awareness, prior planning, and perceptions of the evidence base, rather than logistical or resource-related constraints. Addressing these core issues, through enhanced dissemination of emerging telehealth research, curriculum dialogue, and faculty development initiatives, may have the greatest impact on increasing academic readiness to adopt telehealth teaching within rehabilitation programs.

Clinicians and academics were compared in terms of their familiarity with telehealth, its use in clinical practice, and their perceptions of its role in education (Table 5). Statistically significant differences were observed between the two groups. Academics were more likely than clinicians to support the inclusion of telehealth in the undergraduate curriculum, as indicated by a significant Chi-square result (χ²(2) = 12.49, *p* = .002) with a small-to-moderate effect size (Cramer’s V = 0.25).

Similarly, academics showed stronger endorsement for providing undergraduate telehealth training (χ²(2) = 22.15, *p* < .001), with a moderate effect size (Cramer’s V = 0.33). These effect sizes suggest that the differences between clinicians and academics are not only statistically significant but also meaningful in practical terms, especially regarding attitudes toward training and curriculum development.

**Table 5:** Comparison between academic and clinician groups regarding their familiarity with, use of, and perceptions of telehealth

Characteristics	Study groups		Total *N* (%)	*P* value	Φ/Cramer’s V
	Practitioners *N* (%)	Academics *N* (%)			
Are you familiar with the term “telehealth”?					
No	10 (6.9)	6 (10.7)	16 (8.0)	0.39	0.06
Yes	134 (93.1)	50 (89.3)	184 (92.0)		
Have you ever attended a lecture, program or workshop on telehealth?					
No	71 (49.3)	29 (51.8)	100 (50.0)	0.87	0.02
Yes	73 (50.7)	27 (48.2)	100 (50.0)		
Have you ever presented a talk about telehealth?					
No	109 (75.7)	37 (66.1)	146 (73.0)	0.21	0.09
Yes	35 (24.3)	19 (33.9)	54 (27.0)		
Have you conducted research related to telehealth?					
No	117 (81.2)	44 (78.6)	161 (80.5)	0.69	0.03
Yes	27 (18.8)	12 (21.4)	39 (19.5)		
Self-perception of the inclusion of telehealth as a course to be taught in the curriculum of Rehabilitation programs					
No	11 (7.6)	3 (5.4)	14 (7.0)	0.002*	0.25**
Yes	107 (74.3)	53 (94.6)	160 (80.0)		
Not sure	26 (18.1)	0 (0.0)	26 (13.0)		
Self-perception of the need to teach/train telehealth to undergrad students					
No	5 (3.5)	10 (17.9)	15 (7.5)	< 0.001*	0.33**
Yes	111 (77.1)	46 (82.1)	157 (78.5)		
Not sure	28 (19.4)	0 (0.0)	28 (14.0)		
Total	144 (72.0)	56 (28.0)	200 (100.0)		

*P-value is statistically significant at ≤ 0.05

**Cramér’s V is used where applicable

Φ indicates Phi as the effect size for 2 × 2 tables

## Discussion

Telehealth has evolved from a supplementary tool into a mainstream mode of healthcare delivery, transforming accessibility and efficiency across disciplines [3, 29]. In Saudi Arabia, this transition represents more than a technological advancement; it is part of a broader systemic change aligned with Vision 2030’s commitment to digital transformation and equitable healthcare access [4]. Within this national movement, integrating telehealth into rehabilitation sciences curricula becomes both a strategic and educational imperative to prepare graduates for digitally mediated care environments. This study is among the earliest empirical efforts within the Saudi context to explore how academics and clinicians perceive the integration of telehealth into rehabilitation sciences curricula.

### Perspectives on telehealth: academics vs. clinicians

Both academics and clinicians demonstrated strong support for embedding telehealth education into undergraduate rehabilitation programs. This finding aligns with global literature indicating that telehealth competency is increasingly regarded as essential for contemporary healthcare practice rather than an optional skill [30, 31]. The alignment between the current findings and prior global literature underscores the universality of this educational need across healthcare systems. Moreover, this shared perspective not only validates the current momentum toward integrating telehealth into curricula but also signals an optimistic trajectory for its ongoing development. Although this finding suggests a generally positive attitude toward telehealth among both academics and clinicians, it should be interpreted with caution. Because the study uses a cross-sectional design, the results may be vulnerable to sample biases, particularly if individuals with more favorable views are more inclined to participate. To strengthen the generalizability of these findings, future research should draw from more diverse samples or employ longitudinal designs to determine whether similar levels of support are evident across the broader profession.

Interpreting the findings through TAM clarifies how perceived usefulness and ease of use shape stakeholders’ receptiveness to telehealth; when considered alongside digital-pedagogy frameworks such as TPACK and SAMR, the results underscore that effective curriculum integration requires not only positive attitudes but also faculty capacity to implement technology-enhanced instruction.

Clinicians with prior exposure to telehealth, through practice, workshops, or research, expressed stronger support for curriculum integration. This aligns with the TAM assertion that experience shapes perceptions of usefulness and ease of use [19]. Prior exposure enhances familiarity, reduces perceived barriers, and builds confidence in delivering care through virtual platforms [32, 33]. The findings suggest that experiential learning plays a key role in shaping telehealth readiness underscoring the importance of continuing professional development and simulation-based training.

A distinct divide between academics and clinicians emerged, reflecting broader differences in their roles within technology adoption and curriculum design. Academics’ perspectives tended to be shaped by pedagogical frameworks and educational transformation agendas, whereas clinicians’ views are grounded in immediate service-delivery realities. This divergence may also relate to differences in interprofessional identity development. The concept synthesis of Interprofessional Identity (IPI) by Cantaert et al. [34] suggests that professional perspectives evolve through exposure to collaborative and constructive learning environments. Additionally, literature on knowledge translation highlights that the effective integration of innovation in clinical practice is often hindered by contextual, organizational, and cultural barriers, which can reinforce this divide [35, 36]. Academics’ stronger advocacy for telehealth integration may stem from their engagement with competency-based education frameworks and curriculum governance. Conversely, clinicians’ more cautious approach often arises from practical constraints, such as demanding workloads, limited staffing, and institutional policies that prioritize service delivery over ongoing collaborative education and innovation [36]. This pattern echoes findings from other contexts where educators act as early adopters, whereas practitioners adapt more slowly due to logistical barriers [37, 38].

Bridging this gap requires sustained collaboration between academic programs, clinical sites, and professional associations. Drawing on knowledge translation frameworks, joint committees that co-develop telehealth competencies and field-training guidelines may ensure that curricula remain both pedagogically sound and clinically relevant. Furthermore, encouraging interprofessional learning can improve collaboration and facilitate telehealth integration in education and practice. Future research would benefit from qualitative investigations to explore the underlying drivers of this divide more deeply, especially through the lens of knowledge translation and professional identity formation.

### Academics’ readiness and curricular implications

Among academics, the high endorsement of telehealth integration (over 85%) signifies a strong readiness for curricular innovation despite its limited current presence in rehabilitation programs. Academics who engage in both teaching and clinical practice often serve as early adopters of innovation and recognize telehealth as an evolving competency required for graduate employability [37]. This readiness aligns with the broader calls by professional bodies such as the AAA and ASHA, which advocate for the inclusion of telepractice competencies in professional education [22, 23].

Faculty development is crucial to achieving this transformation. One of the reported challenges in implementing telehealth education is the limited experience and training of educators in delivering telehealth education and clinical supervision [39]. Findings from this study indicated that academics frequently cited a lack of awareness of research supporting this approach as key obstacles to its integration (see Fig. 4). Within this context, the TPACK framework offers valuable guidance for understanding the blend of knowledge educators need to successfully incorporate telehealth into their teaching. Specifically, TPACK emphasizes that effective integration requires educators to combine technological proficiency, pedagogical strategies, and deep content expertise, enabling the meaningful adoption of new technologies in their practices [24].

To address this, national universities should consider launching comprehensive faculty upskilling programs that are aligned with Vision 2030’s objectives for digital capacity building and the accreditation standards for rehabilitation education. Such initiatives would empower educators to confidently deliver telehealth-related content, thereby ensuring consistent, high-quality instruction across rehabilitation disciplines.

In addition to training needs, successful integration of telehealth curricula might require broader institutional readiness and change management frameworks. Implementation readiness is a key component of organizational success in adopting new practices [40]. It encompasses factors such as leadership engagement, the availability of resources (e.g., infrastructure, funding, and training), access to knowledge, and a supportive implementation climate [40]. In our context, the readiness of academic institutions to adopt telehealth education may be shaped by factors beyond faculty expertise which include administrative commitment, investment in digital infrastructure, and alignment with national digital health strategies. Upon review, the selected text is free from significant spelling errors or obvious typographical mistakes. All terminology and names (such as AAA, ASHA, TPACK, Vision 2030) are spelled correctly.

### Barriers and systemic challenges

The Pareto analysis revealed that the main barriers reported by academics included the lack of prior discussion about telehealth course inclusion, limited awareness of supporting research, and uncertainty regarding its relevance and evidence base. To the best of our knowledge, there is limited literature specifically investigating the barriers to integrating telehealth into educational curricula, particularly within allied health and rehabilitation programs. Therefore, our findings are compared to studies that have examined more general barriers to telehealth integration in clinical practice settings. Challenges that have been reported in Saudi and international rehabilitation contexts, where telehealth adoption is hindered by insufficient bandwidth, outdated equipment, and inadequate administrative support [32, 38]. In Saudi Arabia, a recent systematic review identified major obstacles such as limited access to telehealth technologies, low awareness and knowledge among healthcare professionals, and the substantial financial investment required for infrastructure development [38]. Another study noted that the reassessment of existing regulations and policies, especially in the post-COVID-19 landscape, is essential for enabling widespread telehealth adoption across healthcare systems [41].

By looking specifically at the educational dimension of telehealth integration, our study complements the findings of a recent scoping review on telehealth training in allied health disciplines [18]. While that review reported generally positive outcomes from varied teaching approaches, it also underscored a lack of standardized educational models and emphasized the importance of defining explicit telehealth competencies within curriculum design. In our study, the primary barrier identified was the lack of prior discussion, which reveals a deeper issue of institutional inertia and insufficient engagement with digital pedagogy. To address this, we recommend national and institutional efforts such as inter-university task forces, academic symposia, and faculty development workshops to stimulate discussion. These efforts will facilitate the sharing of best practices and help convert increased awareness into unified curriculum reform that is closely aligned with telehealth competencies.

### Educational and professional recommendations

Both academics and clinicians valued the inclusion of telehealth in rehabilitation curricula, with more than 78% of respondents reporting positive perceptions toward offering telehealth courses and training. Integrating telehealth into the curriculum supports equitable service delivery, enhances interprofessional collaboration, and strengthens digital, ethical, and communicative competencies among students [42]. Early curricular exposure also facilitates interprofessional learning, allowing students across rehabilitation disciplines to collaborate in simulated virtual-care scenarios.

To ensure sustainability, academic leaders should:

- Develop national telehealth competency frameworks specific to rehabilitation disciplines.
- Invest in faculty training and mentorship to enhance teaching confidence and skill transfer.
- Incorporate simulation-based telehealth experiences into laboratory and clinical courses.
- Collaborate with clinical partners to integrate telehealth competencies into internship evaluation criteria.

### Theoretical and strategic context

From a theoretical standpoint, the findings resonated with the TAM [19], which posits that perceived usefulness and ease of use shape attitudes toward technological adoption. Academics and clinicians with prior exposure, those who have already perceived telehealth’s value, were more likely to advocate for its integration.

Strategically, these findings aligned with Saudi Vision 2030’s digital health transformation agenda, which calls for collaboration between the Ministry of Health, the Ministry of Education, and universities to standardize digital competencies across healthcare disciplines. Establishing national telehealth education standards would formalize expectations for graduate preparedness and enhance the global competitiveness of Saudi rehabilitation programs.

### Limitations and future directions

This study’s cross-sectional design limits causal inference and reflects perspectives only at a single point in time. Voluntary online participation introduces response bias, potentially favoring individuals who are more digitally literate or who already hold positive perceptions of telehealth. As a result, support for telehealth integration may be overestimated. The use of convenience sampling further constrains the representativeness of the sample, and certain subgroups were disproportionately represented, including a higher proportion of female participants, clustering of academics from specific universities, and uneven distribution across specialties. These imbalances may restrict the generalizability of the findings to all rehabilitation professionals in Saudi Arabia.

In addition, although internal consistency was acceptable for exploratory research, one subscale (barriers) demonstrated lower reliability (α = 0.63). Consequently, findings related to perceived barriers should be interpreted with caution, and future research should refine these items to enhance measurement precision.

Future research could mitigate these limitations by employing stratified or probability-based sampling approaches to ensure broader representation across disciplines, institutions, and regions. Incorporating targeted recruitment strategies, such as direct institutional invitations or incentivized participation, may also reduce self-selection bias and encourage participation from individuals with varying levels of digital confidence. Longitudinal and mixed-methods designs could further enrich understanding of how telehealth education influences competencies and attitudes over time.

## Conclusions

This study offers one of the earliest empirical examinations in Saudi Arabia of how academics and clinicians view the integration of telehealth education within undergraduate rehabilitation curricula. The findings indicate broad agreement that telehealth competency is essential for preparing future rehabilitation graduates; however, they also reveal a persistent gap between educational readiness and actual implementation, driven largely by insufficient technological infrastructure and the absence of standardized training pathways. By situating stakeholder perspectives within established technology-adoption and digital-education frameworks, this study provides a theoretically grounded direction for embedding telehealth competencies into rehabilitation curricula in Saudi Arabia. To effectively shift telehealth from an optional adjunct to a core educational requirement, the proposed roadmap must prioritize the development of a national telehealth competency framework for rehabilitation disciplines, mandatory faculty upskilling programs to ensure confident delivery and supervision, institutional investment in standardized simulation infrastructure, and sustained academic-clinical partnerships to co-develop field-training guidelines that close the current implementation gap.

Taken together, these findings provide a foundational evidence base to inform national curriculum reform and digital health workforce development in Saudi Arabia. By aligning rehabilitation education with national digital health priorities, including Vision 2030, this study supports capacity building at the academic and clinical levels and offers direction for policy-aligned implementation of telehealth education.

## Supplementary Information

Supplementary Material 1.

Supplementary Material 2.

## Data Availability

The data supporting the findings of this study are available from the corresponding author upon reasonable request.

## Abbreviations

AAMC: Association of American Medical Colleges
AAA: American Academy of Audiology
ASHA: American Speech-Language-Hearing Association
