# Supplementary material for: Academics’ and clinicians’ perspectives on telehealth integration in Saudi rehabilitation education
Source: BMC Med Educ. 2026 Jan 24;26:297. doi: 10.1186/s12909-026-08639-4 (PMC12914992; doi:10.1186/s12909-026-08639-4)
Supplement: Supplementary file 1 — Supplementary Material 1. [file 12909_2026_8639_MOESM1_ESM.pdf]

Perspectives of Health Academics in Saudi Universities towards the Potential Inclusion of Telehealth in Academic Programs

1. Overview

1. Do you agree to participate in the study?

☐ Yes

☐ No

2. General information

2. Age

☐ 21-30 years old

☐ 31-40 years old

☐ 41-50 years old

☐ 51-60 years old

☐ More than 60 years old

3. Gender

☐ Male

☐ Female

4. Nationality

☐ Saudi

- [ ] Non-Saudi

5. At what university do you work?

[ ]

6. Speciality

- [ ] Physiotherapy
- [ ] Occupational therapy
- [ ] Respiratory therapy
- [ ] Speech and language pathology
- [ ] Audiology
- [ ] Other Rehabilitation speciality (please specify):

7. Academic rank

- [ ] Lecturer
- [ ] Assistant professor
- [ ] Associate professor
- [ ] Professor

8. For how many years have you been teaching?

- [ ] Less than 2 years
- [ ] 2-5 years
- [ ] 6-10 years
- [ ] More than 10 years

3. Familiarity with Telehealth

9. Do you feel comfortable using technology?

- [ ] Yes
- [ ] No

10. Are you familiar with the term "Telehealth"?

- [ ] Yes
- [ ] No

11. Have you ever attended a lecture, program or workshop on Telehealth?

- [ ] Yes
- [ ] No

12. Have you ever taught a course/topic related to Telehealth?

- [ ] Yes
- [ ] No

13. Have you conducted a research related to Telehealth?

- [ ] Yes
- [ ] No

14. Do you practice clinically? (either at the university's clinics or at an outside clinic)

- [ ] Yes
- [ ] No

4. Telehealth clinical practice

15. Do you use Telehealth in your practice?

- ☐ Yes
- ☐ No

5. Teaching Telehealth

16. Do you think the skills for providing Telehealth services should be taught to undergraduate students and clinical training should be provided to them?

- ☐ Yes
- ☐ No

17. Do you think "Telehealth" should be included in the Rehabilitation curriculum of undergraduate students?

- ☐ Yes
- ☐ No
- ☐ Not sure

18. Does your program have an academic course in Telehealth application?

- ☐ No
- ☐ Yes, we have it as an independent elective course
- ☐ Yes, we have it as an independent mandatory course
- ☐ Yes, we have it as a mandatory topic within some of the program courses
- ☐ Other (please specify): ____________________

6. Willingness of inclusion

19. Is there a plan in your program to include Telehealth teaching/training in the curriculum?

- [ ] No
- [ ] Yes, within a year
- [ ] Yes, within 3 years
- [ ] Yes, but not sure when
- [ ] Other (please specify):

7. Unwillingness of inclusion

20. In your opinion, what is the reason for the unwillingness to include Telehealth in your program curriculum?

- [ ] The inclusion of a Telehealth course was not discussed before in the program
- [ ] Telehealth is not recommended/useful for our speciality
- [ ] There is no benchmark for teaching such a course
- [ ] Lack of awareness of researches supporting this approach
- [ ] There is insufficient research work to support this approach
- [ ] Lack of faculty members who could teach and/or provide clinical training of such a course
- [ ] Lack of technology resources
- [ ] Not interested
- [ ] Other (please specify):
