# Supplementary material for: Academics’ and clinicians’ perspectives on telehealth integration in Saudi rehabilitation education
Source: BMC Med Educ. 2026 Jan 24;26:297. doi: 10.1186/s12909-026-08639-4 (PMC12914992; doi:10.1186/s12909-026-08639-4)
Supplement: Supplementary file 2 — Supplementary Material 2. [file 12909_2026_8639_MOESM2_ESM.pdf]

Perspectives of the Health Practitioners in Saudi Arabia towards the Potential Inclusion of Telehealth in Academic Programs

1. Overview

1. Do you agree to participate in the study?

- [ ] Yes
- [ ] No

2. General information

2. Age

- [ ] 21-30 years old
- [ ] 31-40 years old
- [ ] 41-50 years old
- [ ] 51-60 years old
- [ ] More than 60 years old

3. Gender

- [ ] Male
- [ ] Female

4. Nationality

- [ ] Saudi

- [ ] Non-Saudi

5. Place of work

- [ ] Public hospital
- [ ] Private hospital/clinic
- [ ] Both public/private
- [ ] Ministry of Health
- [ ] Other (please specify):

6. Speciality

- [ ] Physiotherapy
- [ ] Occupational therapy
- [ ] Respiratory therapy
- [ ] Speech and language pathology
- [ ] Audiology
- [ ] Other (please specify):

7. Rank

- [ ] Specialist
- [ ] Senior specialist
- [ ] Consultant

8. For how many years have you been practicing?

- [ ] Less than 2 years
- [ ] 2-5 years

- [ ] 6-10 years
- [ ] More than 10 years

9. Have you ever collaborate to teach any course either theoretical or practical at a university?

- [ ] Yes
- [ ] No

3. Familiarity with Telehealth

10. Do you feel comfortable using technology?

- [ ] Yes
- [ ] No

11. Are you familiar with the term "Telehealth"?

- [ ] Yes
- [ ] No

12. Have you ever attended a course, workshop or program on Telehealth?

- [ ] Yes
- [ ] No

13. Have you ever presented a talk about Telehealth?

- [ ] Yes
- [ ] No

14. Have you ever conducted a research on Telehealth?

- [ ] Yes
- [ ] No

15. Do you use Telehealth to deliver services in your clinic?

- [ ] Yes, regularly
- [ ] Yes, sometimes
- [ ] No

4. Telehealth system

16. What system do you use to provide the service?

- [ ] Hospital system
- [ ] Commercial system
- [ ] Not sure
- [ ] Other (please specify):

5. Teaching Telehealth

17. Do you think Telehealth practice is useful for your speciality?

- [ ] Yes
- [ ] No
- [ ] Not sure

18. Do you think the skills need for conducting Telehealth services should be taught to undergraduate students and clinical training should be provided to them?

- [ ] Yes

- [ ] No
- [ ] Not sure

19. Do you support the inclusion of Telehealth as a course to be taught in the curriculum of Rehabilitation programs?

- [ ] Yes
- [ ] No
